# Editorial: Cancer Prevention: Targeting Premalignant Epithelial Neoplasms in the Era of Cancer Immunotherapy and Vaccines

**DOI:** 10.3389/fimmu.2022.924099

**Published:** 2022-05-24

**Authors:** Nicolas Çuburu, Olivera J. Finn, Sjoerd H. Van Der Burg

**Affiliations:** ^1^ Laboratory of Cellular Oncology, Center for Cancer Research, National Cancer Institute (NIH), Bethesda, MA, United States; ^2^ Department of Immunology, School of Medicine, University of Pittsburgh, Pittsburgh, PA, United States; ^3^ Department of Medical Oncology, Leiden University Medical Center, Leiden, Netherlands

**Keywords:** vaccine, immunotherapy, antigen discovery, animal model, premalignant lesion, tumor microenvironment

Prevention of cancer is an essential intervention to reduce the cancer burden globally and public health measures targeting etiological factors such as tobacco consumption or oncogenic viruses as well as screening of early lesions have been extremely successful. Indeed, prophylactic vaccination against oncogenic human papillomavirus and hepatitis B virus have paved the way for immune-based interventions for the prevention of cancer (1).

Cancer immunotherapy was named the breakthrough of the year 2013 by Science Magazine; a decade later it has truly revolutionized the field of oncology (2). Advances in immuno-oncology are focused on late-stage metastatic cancers with the most urgent needs and highest potential to improve patients’ immediate health. Remarkably, cancer immunotherapies such as immune checkpoint blockade or adoptive cell transfer have been associated with complete and durable cures, likely a consequence of the induction of long-lived anti-cancer immune responses. However, treatments of advanced cancers must also overcome powerful mechanisms of immune resistance in tumors and severe systemic adverse events, thereby preventing a higher therapeutic index.

Inspired by these successes, harnessing the immune system for the prevention and treatment of cancer could have a tremendous impact on cancer burden at a population level as well as providing immediate benefit to patients at high risk of developing cancer. To unleash this potential, the development of new diagnostics and immune interventions requires a deep understanding of the continuum of cellular and molecular modifications that often accumulate through many decades of cancer progression (3). The key factors could now begin to be identified at the early premalignant stages of cancer. This collection of original research and review articles covers emerging concepts in the field of premalignant lesions biology and offers a glimpse at possible immune-based interventions, including vaccines and combination therewith, to prevent their progression to cancer.

Initiatives to categorize systematically the immune and genetic landscapes of benign lesions such as the precancer genome atlas could provide a rationale for new interventions (4). A recent analysis of the progression of lung squamous cell carcinoma (SCC) showed that local immune activation and immune suppression occur in premalignant lesions but is limited compared to immune modulation in high grade lesions and SCC (5). In this issue, Rangel et al. provide a comprehensive overview of HPV-negative head and cancer progression and how genetic alterations, notably TP53, CDKN2A and NOTCH1, lead to accumulation of macrophages, MDSCs and Treg in the microenvironment of developing lesions. Chadwick et al. provide evidence of the early contribution of TNF-α in the recruitment of inflammatory cells and progression of oropharyngeal squamous cell carcinoma (OPSCC) in a small cohort of patients. Using a chemically induced (4-NQO) OPSCC animal model they further demonstrate that targeting TNF-α could indeed prevent the progression of OPSCC in mice. Roudko et al. provide a very informative review on the genetic basis of Lynch syndrome caused by mutations in DNA mismatch repair (dMMR) resulting in genome instability. In addition, they discuss the status of current vaccines targeting neoepitopes associated with genome instability in microsatellite coding regions. Zachariah et al. provide a comprehensive review on the various types of premalignant breast cancer lesions and discuss the integration and advantages of targeted vaccines, notably HER2, in the context of standard of care and chemoprevention. Jacqueline et al. investigated the interaction between breast cancer cell lines that recapitulate the continuum of cancer progression, with macrophages *in vitro*. These elegant coculture experiments indicate that cell lines corresponding to normal epithelia or early premalignant phenotypes are not recognized by macrophages, but those corresponding to late premalignancy or advanced cancer phenotype cause pronounced activation of macrophages. They further identify surface markers, Annexin-A1 and CEACAM1, that are responsible for the interaction between breast premalignant and tumor cells and macrophages.

Transplantable syngeneic tumor models have been instrumental to establish proof of principle for therapeutic interventions against advanced cancer and to understand mechanisms of immune control and resistance to therapies. However, due extremely fast growth and profound genomic alterations, these models cannot fully recapitulate the slow development of cancer in situ. This collection offers excellent examples of animal models that can be leveraged for better understanding of premalignant lesions and cancer progression and to evaluate novel candidate vaccines or therapeutic interventions. Neckerman et al developed an adenoviral vector vaccine platform to be evaluated in a non-human primate model of persistent papillomavirus infection. Rangel at al. and Chadwick et al. used a chemically induced mouse model to study early immune modulation in OPSCC. Finally, Corulli et al. report two chemically induced (AOM) and transgenic APCmin transgenic colorectal cancer models for the evaluation of therapeutic vaccination against TAA. All these are models of spontaneous development of cancers and therefore are technically more challenging than classic syngeneic transplantable models as they require longer time to develop and have a variable penetrance.

The identification of protective antigens is critical to the development of vaccines for the treatment of premalignant neoplasia and to prevent cancer progression, Corulli et al. demonstrate by serology and T cell IFN-γ assays, that in colorectal cancer and adenomatous polyps overexpressed proteins CDC25B, COX2, RCAS1, and FASCIN1 are *bona fide* tumor associated antigens. In murine models of colorectal cancer, they demonstrate proof of principle of cancer prevention after vaccination by showing that CDC25B, COX2 and RCAS1 immunization leads to reduced tumor formation. These results provide a rationale for the identification, development, and use of TAA-based vaccines against colorectal cancer and its premalignant precursors. Understanding the genomic instability in microsatellite coding regions led to the identification of recurrent frameshift mutations encoding potentially protective neoantigens. Notably, preclinical studies in murine model of Lynch syndrome have shown improved survival and reduced tumor burden and a frame shift vaccine based on long peptide and CpG as adjuvant (6). Such vaccine could be the first off-the-shelf cancer vaccine to intercept colorectal cancer and other epithelial cancers at early stages before extensive immune suppression is established as an extrinsic resistance mechanism and before the acquisition of driver mutations in APC, BRAF and TP53. Zachariah et al. provide an exhaustive review on breast cancer precursor lesions and an update on current targeted therapies driver antigens such as HER-2 and breast antigens such as MUC-1, Lactaglobin and mammaglobin. Accumulating evidence of a good safety profile of dendritic cells vaccines targeting HER2 compared to chemoprevention offers an opportunity to intercept the development of invasive breast cancer at the early stages before immunoediting occurs and durable benefit may be obtained. Such approach if successful could provide safer alternative to chemoprevention at the DCIS or premalignant stages.

Cancers caused by infections contribute to 15% of the global cancer burden. The foreign nature of oncogenic viruses has allowed the development of powerful prevention tools to reduce the burden of virus-related cancers. For instance, HPV is a family of epitheliotropic virus with circular double stranded DNA and the main causative agent of cervical cancer and other epithelial cancers. Antibodies against the L1 capsid protein of HPV can effectively prevent infection and subsequent epithelial cell transformation driven by the viral oncogenes E6 and E7 which is at the basis of the current HPV VLP prophylactic vaccines. Huang et al. report a new humanized monoclonal antibody targeting the major capsid protein L1 of the oncogenic HPV type 18 which they propose constitute a novel type of microbicide. The implementation of such approach remains to be defined in the context of a widespread availability of highly efficacious prophylactic HPV vaccines as it is likely that such antibodies would be limited to prophylactic usage as HPV remains impervious to antibody neutralization once infection is established. In contrast, nonstructural E6 and E7 HPV oncogenes whose primary functions cause the degradation of p53 and Rb are required for cancer progression and persistence. These viral oncogenes represent ideal targets of therapeutic cancer vaccines as they are drivers of cancer progression. Several clinical trials including E6 and E7 antigens as synthetic long peptides or nucleic acid platform against cervical and vulvar intraepithelial neoplasia have shown therapeutic benefit and regression of premalignant lesions (7, 8). Most genital HPV infection, however, are naturally cleared, and only persistent infection can lead to the development of premalignant lesions. Neckermann et al. propose a vaccine targeting nonstructural viral antigens E1 and E2 that are highly expressed during persistent HPV infection but lost as the lesions progress. They designed a vaccine candidate targeting the E1/E2 viral antigens to be evaluated in a Macaca fascicularis papillomavirus, a model of persistent natural infection that leads to the development of local lesions.

The recent successes of SARS COV2 mRNA vaccine showed that disruptive technologies can have a tremendous impact on public health. Beyond antigen characterization, this issue offers a glimpse at potential cancer vaccine candidates in terms of platforms, modalities, and adjuvants. Nekerman et al. report an adenoviral vector that can be delivered systemically or mucosally to target specifically mucoepithelial premalignant lesions. Corulli et al. report a vaccine platform based on selected MHC class 2 peptides given with water-in-oil-in-water adjuvant (CFA/IFA). In this model, vaccine efficacy relies on CD8+ T cells suggesting epitope spreading against antigens that were not included in the vaccine (9). Roudko et al. offers an overview of current vaccines against frame shift mutations in Lynch syndrome which include synthetic long peptides with a water-in-oil-in-water adjuvant (Montanide) or viral vectors (MVA, adenoviral vectors). Zacharia et al. offers a thorough overview of vaccine platforms targeting breast cancer with the potential to be repurposed against early lesions. Of note dendritic cells pulsed with HER2 showed excellent safety profile and efficacy, akin to the Sipuleucel-T vaccine, which is the first approved cancer for castrate resistant metastatic prostate cancers. Finally, in addition to HPV and HBV, vaccines against EBV and hepatitis C could have a profound impact in the prevention of nasopharyngeal carcinoma, Non-Hodgkins lymphoma and hepatocellular carcinoma. In addition, a Merkel cell polyomavirus vaccine targeting viral oncogenes is being evaluated for advanced Merkel cell carcinoma, but it could also prevent recurrence and metastasis if used in combination with surgical removal of early lesions.

With the increasing number of cancer epitopes that are validated experimentally, it becomes important to catalogue and curate these large datasets in order to facilitate the dissemination of information to the scientific community. Building on the successes of Immune Epitope Database (IEDB) in the field of autoimmunity and infectious diseases, Kosaloglu-Yalçin et al. present Cancer Epitope Database and Analysis Resource (CEDAR), a new platform that will offer soon a curated dataset of cancer epitopes that can be used to assess and refine prediction algorithms relevant to cancer antigens. This new dataset together with machine learning algorithm has the potential to integrate new relevant parameters and to build more powerful algorithm to predict actionable tumor antigens (10).

To conclude, remarkable efforts have been made to identify tumor associated antigens, characterize the premalignant and tumor immune microenvironments, and to develop vaccine platforms and adjuvants. This Research Topic offers an updated perspective on how to effectively use these advances to target premalignant lesions as a way to cancer prevention. Targeting early-stage of cancer development presents the advantage of targeting lesions when they are most likely to regress. Albeit, even at this stage, differences in the immune landscape may affect clinical outcome of different forms of immunotherapy (3). Important questions will need to be answered in the near future. For instance, can we identify markers of early lesions for most cancers and what would be the protective antigens? Can we develop therapeutics that tip the risk/benefit ratio in favor of intervention? What will be the long-term efficacy and safety profiles of vaccines targeting tumor associated antigens in terms of autoimmunity? How to integrate such preventative vaccines into routine preventative cancer screening and treatment? Because screening offers opportunity to effectively treat some developing lesions by surgical excision, it is likely that the impact of vaccines targeting premalignant lesions will be maximal either as neoaduvant therapy to prevent or downscale the surgical procedure (and as such the complications associated with it) or as adjuvant therapy to prevent later recurrences. Finally, the reduced cost and ease to deploy vaccines could have a tremendous impact in low resource settings where access to surgical procedures can be limited.

## Author Contributions

All authors listed have made a substantial, direct, and intellectual contribution to the work and approved it for publication.

## Conflict of Interest

The authors declare that the research was conducted in the absence of any commercial or financial relationships that could be construed as a potential conflict of interest.

## Publisher’s Note

All claims expressed in this article are solely those of the authors and do not necessarily represent those of their affiliated organizations, or those of the publisher, the editors and the reviewers. Any product that may be evaluated in this article, or claim that may be made by its manufacturer, is not guaranteed or endorsed by the publisher.
